# New insights into semen separation techniques in buffaloes

**DOI:** 10.3389/fvets.2023.1347482

**Published:** 2024-01-10

**Authors:** Crina Raluca Andrei, Florin Petrișor Posastiuc, Nicolae Tiberiu Constantin, Ioan Liviu Mitrea

**Affiliations:** ^1^Faculty of Veterinary Medicine of Bucharest, University of Agronomic Sciences and Veterinary Medicine, Bucharest, Romania; ^2^Faculty of Veterinary Medicine, Ghent University, Merelbeke, Belgium; ^3^Research and Development Institute for Bovine Balotești, Balotești, Romania

**Keywords:** assisted reproductive technology, buffalo, semen separation methods, centrifugation, filtration

## Abstract

Male infertility is frequently caused by idiopathic or unexplained reasons, resulting in an increase in demand for assisted reproductive technologies. In buffaloes, more than in other animals due to reproductive hardiness, successful fertilization needs spermatozoa to effectively transit the female reproductive system to reach the oocyte. This mechanism naturally picks high-quality sperm cells for conception, but when artificial reproductive technologies such as *in vitro* fertilization, intracytoplasmic sperm injection, or intrauterine insemination are utilized, alternative techniques of sperm selection are necessary. Currently, technology allows for sperm sorting based on motility, maturity, the lack of apoptotic components, proper morphology, and even sex. This study provides current knowledge on all known techniques of sperm cell sorting in buffaloes, evaluates their efficiency, and discusses the benefits and drawbacks of each approach.

## 1 Introduction

The domestic buffaloes are a vital livestock resource with soaring economic importance especially for developing countries. The world’s buffalo population grew by 0.8% annually between 1991 and 2002 and by 1.3% annually between 2002 and 2017, suggesting a recent rise in interest in buffalo husbandry (1). However, reproduction techniques applied in this sector are facing several challenges such as: silent heat (2, 3) and unsatisfactory oestrus detection (4–6), anestrus (2), (7) seasonal infertility (8, 9), longer calving intervals (10, 11), delayed puberty (12, 13) and specific low number of primordial follicles (14) along with the high rate of atresia and apoptosis (13, 15). While these limiting characteristics specific to the female component can be diminished by applying different synchronization protocols (16–18), the use of high-quality semen has to be also addressed for the optimisation of fixed time insemination. Recently, important countries for the buffalo industry have raised concerns regarding the decreased availability of sires with high genetic quality and poor capability of some individuals to qualify for semen collection (19), highlighting the importance of semen selection and preservation in this sector. Along with several enrichment protocols (20–23), sperm separation techniques have emerged as valuable tools in the field of reproductive biology and assisted reproduction. Furthermore, the development of techniques that can effectively separate the motile sperm fraction from the other components of semen is essential to the success of assisted reproductive technology (ART). Reproductive biotechnologies have a unique role in improving livestock herds in contemporary high-efficiency livestock breeding. They also provide global access to new genetics, which benefits biodiversity conservation in animal research (24).

### 1.1 Buffalo semen

Even though both buffaloes and cattle bulls are classified as large ruminants (25), there are some anatomical differences and variations in terms of sperm characteristics, concerning the volume (mL) (2.958 ± 0.18, respectively 4.038 ± 0.22), concentration (1.678 × 10^9^, respectively 1.736 × 10^9^) (26), pH (6.79 ± 0.01, respectively 6.80 ± 0.01), sperm density (2.63 ± 0.08, respectively 2.57 ± 0.07) and abnormal sperm (7.64 ± 0.36, respectively 8.86 ± 0.38) (27). Moreover, the capacity to fertilize oocytes *in vitro* and the subsequent *in vitro* growth of embryos vary significantly between buffalo and cattle bulls’ sperm (28). The normal colour of buffalo semen is creamy white with a clean aspect (29). The mean volume of the ejaculate in Romanian buffalos, already genetically characterized (30) was found to be 4.07 (± 0.02) mL (29), which is less than in other indigenous buffalo breeds: Banni 4.09 (± 1.59), Bhadawari 4.11 (± 1.57), Jaffarabadi 5.10 (± 1.80), Murrah 4.48 (± 1.87), Pandharpuri 4.79 (± 1.80), Surti 4.68 (± 1.73) (31). An important difference was observed between the sperm pH of Romanian buffalo (29) that was 5.81 (± 0.06) and the swamp buffalo bulls in Thailand which was 7 during winter and summer and 6.9 during the raining season (32). The mean motility sperm percentage in Romanian buffalo was 71.5 (± 0.03) (33), being slightly lower than in Asian buffalo (75.2 ± 1.3) (32). In contrast to the motility, sperm concentration in the local buffalo (33) is higher (1.65 × 10^9^ per mL) than that of Thai (1.1 × 10^9^ per mL) (32).

### 1.2 Sperm separation methods

The swim-up method (SU) along with the density gradient centrifugation (DGC) represent the most commonly used separation methods for livestock ART applications (33). Both of them are able to quickly and economically eliminatethe low-quality spermatozoa, unwanted cells, and bioactive particles, resulting in the isolation of longer telomere spermatozoa (34) that can represent an indicator of unimpaired spermatogenesis (35).

Glass wool filtration (GWF) uses tightly packed glass wool fibers to separate immotile sperm cells from motile spermatozoa, the self-propelled mobility of the spermatozoa and the filtering effect of the glass wool being the fundamental components of this sperm separation technology (36).

Sephadex gel filtration (SGF) demonstrates the ability to confine spermatozoa with compromised acrosomes or damaged membranes inside a particular dextran gel column (37).

Magnetic-activated cell sorting (MACS) is one of the most efficient methods of isolating the spermatozoa with deteriorated membranes (apoptosis) in order to provide a high-quality sperm fraction (38). This paper depicts the proposed sperm separation methods utilized in buffaloes along with their effect on semen quality and ART success.

### 1.3 Literature research

A systematic exploration of the literature was conducted using Pubmed database (1975-2022). The following search strategy was implemented into Pubmed: “semen separation techniques buffalo,” “assisted reproductive technology animals,” “swim-up method buffalo density gradient centrifugation buffalo semen,” “glass wool filtration buffalo,” “sephadex gel filtration buffalo,” and “magnetic-activated cell sorting sperm.”

The electronic search of Pubmed returned 297 papers. Following reading the titles and abstracts, 107 were found to be related to semen assessment and different techniques separation. Tabel 1 provides the descriptive data of all studies for the review highlighting the semen separation methods, species, *in vivo,* and *in vitro* semen evaluation.

## 2 Sperm separation techniques

### 2.1 The density gradient centrifugation method

Different density gradients have been tested, in order to determine their suitability for bovine sperm selection, retrieving higher motility samples with preserved gene expression, acrosomal membrane and DNA integrity (33). Percoll gradient centrifugation relies on the higher density of the nucleus within normal spermatozoa which permits further deposition of the stated in the elevated density region (39). Moreover, it was proven in bovines that highly motile spermatozoa will deposit faster due to the alignment to the centrifugal forces (40).

The ability of Percoll density gradient method to augment X-bearing viable sperm by up to 70% (41, 42) while preserving the sperm membrane and acrosome intact (43), supported the use of density gradient for sexed semen in livestock reproductive biology. In buffaloes, enriched semen after the use of Percoll was trialed for X and Y sperm separation (39, 44, 45) as well, the consequent effect on sperm quality being studied in comparison with filtration techniques (46) or classic swim-up (47).

Through the use of 45 and 90% Percoll solutions, buffalo thawed frozen semen were processed resulting in a low recovery rate with concentrations after centrifugation reaching 4.7 ± 1.5 × 106/mL, which is corresponding to 29.57 times decrease compared to mean original post thaw motility (47). However, the mean motility of the isolated spermatozoa was higher edging 63.1 ± 9.0 %, compared to pre-centrifugation motility rates of 38.5 ± 4.9 % (47). Moreover, significant enhancement of membrane integrity (MI) and acrosome integrity (ACI) were noted (70.5 ± 7.6 % MI; 70.6 ± 11.1 % ACI) (47) (see Table 1).

**Table 1:** Classification of scientific publications on sperm separation techniques according to the method used, the species, and the *in vivo* or *in vitro* method.

Separation method	Species	*In vivo* method [references]	*In vitro* method [references]
The swim-up method	Cattle bull	(48)	
	Buffalo bull		(49) (50) (51) (52)
	Men	(53)	(54) (55) (56) (57) (58)
The density gradient centrifugation method	Men	(53)	(55) (56) (58) (59) (60) (61)
	Stallion		(62)
	Dog		(63)
	Cattle bull		(64)
	Buffalo bull		(65)
The glass wool filtration	Stallion		(66) (67) (68) (69)
	Pony stallion		(66)
	Men		(70) (71)
The sephadex gel filtration	Boar		(72) (73)
	Buffalo bull		(50) (74) (75) (76)
	Stallion		(67)
The magnetic-activated cell sorting	Men	(77) (78)	(79) (80) (81) (58) (82)
	Cattle bull		(83) (51)
	Mouse	(84)	
	Stallion		(62)
	Rabbit		(49)

Similarly, using colloidal suspensions of silica particles, three discontinuous gradient preparations (45% & 95, 65% & 95 and 45%, 65, 95%) were investigated in order to assess the effectiveness of separation (85). For all the gradients formely listed, the values for the motility rates were 77.13 ± 7.9%, 87.38 ± 7.9%, respectively 96.40 ± 1.9% and the recovery rates with concentrations of 15.00 ± 0.8%, 10.46 ± 5.6%, respectively 9.00 ± 4.9. It was observed that the motility rates were higher when using three-layer centrifugation (96.40 ± 1.9%) while the recovery rate was better for the 45-95% formulationpossibly showing more exclusive isolation ability of the three-layered method in terms of motility (85).

The effect of density gradient separation on *in vitro* fertilization (IVF) was surveyed through the oocyte cleavage rate of the obtained samples. Both experiments (47, 85) discussed also the bull effect when interpreting results. This impact might be generated by changes in sperm capacitation across bulls throughout the fertilization process (86), with differences in fertilizing capability dependent on sperm penetration kinetics (87). Spermatozoa that capacitated and fertilized more quickly produced zygotes that cleaved and matured more quickly than those that cleaved later (88). However, cleavage rates varied subsequently to the gradient formulation from 55.6 % for 45-90 Percoll (86) to 69.1 ± 2.04 % for three layered 45-65-95 preparation (86) (Table 2).

**Table 2:** Effect of different colloidal preparations on semen quality and fertilizing ability of buffalo semen.

Formulation (%)	Semen type evaluated	Semen recovery rate (%)	Mean motility (106/mL)	Mean cleavage rate (%)	MI(b) (%)	ACI(c) (%)	Reference
20-40	Fresh	44.68	-	-	12.68 ± 4.63	26.35 ± 6.84	(46)
10-20-30-40-50-60-70	Fresh	-	86.17 ± 0.48(a)	-	82.17 ± 0.98	82.00 ± 0.82	(45)
45 - 90	Frozen	3.38	63.1 ± 9.0	55.6	70.5 ± 7.6	70.6 ± 11.1	(47)
40-80	Frozen	-	86.0 0 ± 1.50	-	30.0 ± 0.6	80.50 ± 2.52	(60)
45 - 95	Frozen	39.68	77.13 ± 7.9	63.6 ± 1.70	-	-	(85)
65 - 95	Frozen	27.67	87.38 ± 7.9	65.1 ± 2.34	-	-	
45 – 65 - 95	Frozen	23.81	96.40 ± 1.9	69.1 ± 2.04	-	-	

Value refers to progressive motility.

MI - Membrane integrity.

ACI - acrosome integrity.

A seven layered preparation (70, 60, 50, 40 30, 20 and 10%) was proposed for fresh, highly mobile (mass motility > + 3 and progressive motility >70%) buffalo semen in order to obtain sexed material (the percentage of female fetuses increased by 66.66%) (45). Secondary to the stated experiments, while selecting X bearing spermatozoa, the protocol was beneficial also for the overall quality of the semen, higher progressive motility, and membrane and acrosome integrity (45).

In terms of morphology, while using Percoll as a 40-80% double layered method on frozen semen, lower abnormalities were observed (7.8 ± 0.7 %) in comparison to the control groups (18.8 ± 1.94 %) and even to other separation techniques such as swim up (8.8 ± 0.57 %) or Sperm/Sperm-Tyrode’s Albumin Lactate Pyruvate (sp-TALP) washing (13.4 ± 1.23 %) (60).

It is noteworthy that there are studies in which volume and density of Percoll gradient used was much higher (2 mL Percoll 90% solution) (44) than in others (0.5 mL Percoll 45% solution) (48). Therefore, it’s possible that the higher height and density of the column provided an additional obstacle to the sperm cells’ movement, causing them to come into prolonged contact with Percoll (44). This might have caused certain changes, like a higher rate of capacitation and an earlier acrosome reaction (89). Thus, it is plausible to hypothesize that the use of Percoll gradients with smaller volumes actually results in lesser acrosomal damages. Therefore, while using density gradients with high volumes in assisted reproduction, we need to take into account the possibility of such acrosomal damage (44). If a double DGC is used, the first DGC in a normal semen readily separates motile sperm, whereas the second DGC leads to the assembly of sperm with a restricted ability to move, including immotile sperm. Therefore, it is more essential and more effective to use a second DGC to separate sperm from inadequate semen samples (56).

### 2.2 The swim-up method

Primarily described in 1984 (90), SU follows a basic principle regarding the capacity of motile spermatozoa to migrate toward a cell-free medium usually placed above the sample (91). The SU’s applicability and effect on sperm quality was assessed in buffaloes, some important traits for ART applications being even superior to other separation techniques (50, 60, 85, 92).

Previous experiments showed that SU separated sperm had superior motility (69.1 ± 8.0 %) and significantly higher MI (77.3 ± 8.9 %) when compared to DGC, but the recovery rates were generally lower (85). Moreover, based on cleavage rate analysis SU proved to be more feasible for IVF (significant differences between cleavage rate and cleavage index *p* < 0.05) (85). Another paper depicted SU as being deficient in ACI preservation, registering a lower percentage of total intact acrosomes in comparison with DGC (68.2 ± 3.21 % vs. 80.5 ± 2.52 %) (60).

In terms of progressive motility, SU was detrimental to different filter separation techniques such as glass wool filtration (GWF) or Sephadex gel filtration (SGF), returning lower values in post-thaw buffalo sperm samples 55.83 ± 1.53 % (SGF 68.33 ± 1.05 %; GWF 65.83 ± 1.54 %) (92). The same pattern was observed when assessing the effect of the three methods on sperm viability and further livability (92). Secondary to oocyte insemination, SU cleavage rates were this time approximatively similar to filter separation techniques, bordering the control samples (SU 21.33 ± 1.94 %; control 21.98 ± 3.00 %) (92). Similar results were reported by older data as well, the recovery rate of motile spermatozoa after SGF being significantly higher than after SU (50).

For the means of sperm sexing, a modified SU method has been validated, the recovery rate for X bearing spermatozoa (5.19 ± 2.04 %) being significantly superior to Y chromosome bearing spermatozoa (0.70 ± 0.15 %) (52). Although MI and AI rates were higher in both X and Y categories when compared to the control, the progressive motility of the non-separated samples was higher (85.00 ± 0.57 % > 76.33 ± 1.11 % X-sorted and 69.67 ± 0.66 Y-sorted) (52). However, those results were obtained prior to freezing, and post thawed samples which were subjects to the modified SU were, in fact, superior in terms of progressive motility (54).

The percentages of DNA fragmentation were 18.30 ± 10.8 in raw samples, 6.6 ± 5.7 after direct SU, 12.9 ± 9.9 after density gradient (DG), 3.7 ± 4.0 after density gradient followed by swim-up (DG-SU), and 4.2 ± 3.8 after pellet SU, the last one being one of the best options in the treatment of semen during IVF/ICSI due to the low cost and reduced time (54).

### 2.3 Filter separation

#### 2.3.1 The glass wool filtration

The protocol of GWF consists in taking a tuberculin syringe or cutting a 1 mL plastic syringe was cut at the 0.6 mL mark and adding 15 mg of glass wool. In order to remove loose glass fibers or any debris, the syringe was flushed with different medium (Ham’s F-10 or TH3) after being positioned perpendicularly in a test tube. An aliquot of the ejaculate was centrifuged to wash it with medium after it had been liquefied. Re-suspended in 1 mL medium, the pellet was laid onto the wet glass wool and allowed to gravity-filter itself without the use of suction or pressure (93, 94).

When spermatozoa are dead or damaged, their plasma membranes alter, which is followed by their binding with glass fibers, producing the desired effect in GWF (95). The type of glass wool used has a direct impact on how well this technique works (36). This method has been proven to be adequate for the recovery of high-quality semen in stallions, results being similar or even better when compared to colloid centrifugation (96). A similar outcome was reported in buffaloes, GWF being able to retrieve actually higher cleavage rates (28.97 ± 4.07 %) than the SU method (21.33 ± 1.94 %), in embryos generated after oocyte insemination (92). Older data states that using GWF more motile spermatozoa may be recovered detrimental to SU (95 ± 3.7 vs. 33 ± 5.5 %) (50). Moreover, the general recovery rate of total and motile spermatozoa was higher for GWF than SGF or SU (92). In addition, a larger proportion of live cells with increased mitochondrial activity and a functioning membrane could be chosen thanks to the usage of glass wool (66).

By combining standard GWF with annexin V binding, its effectiveness can be further enhanced (97). Glass wool filtration, like density gradient centrifugation, uses the entire volume of the ejaculate in comparison to swim-up or migration-sedimentation methods, yielding a far higher total number of motile spermatozoa (36, 70). Furthermore, given its affordability, ease of use, and superior responsiveness to viable spermatozoa of equine semen, its usage ought to be promoted (66).

#### 2.3.2 The sephadex gel filtration

Filtration using Sephadex columns is an additional method of sperm separation, which was trialed in different species including rams (98), bulls (41), buffaloes (49, 76), boars (49) and stallions (67, 74). With this purpose different pore sizes were used ranging from G-10 to G-200, SGF exhibiting the capacity to trap the spermatozoa with damaged membranes or defective acrosomes within the specific dextran gel column (37, 73). Depending on the size of the molecules, there are more types of Gel (G-10, 15, 25, 50, 75, 100, 150, 200), G-10 being for small molecules (<700 Da) and G-200 for larger molecules (5000-250000 Da).

In buffaloes, comparing filtered and unfiltered semen, it has been shown that the percentage of progressive motile sperm and live sperm increased significantly thanks to the use of SGF (49, 74, 76, 99).

Comparative surveys between separation methods or even distinct Sephadex grades were carried out in buffaloes during the last 20 years, the results being still subject to discussion. Even if G-75 has been recommended for wider use in buffaloes (99), some authors considered G-100 as also suitable, being superior in improving semen quality when compared to G-200 or G-15 (37). Moreover, it was proven that the use of G-75 and G-100 columns did not result in significant variations regarding the mean recovery rate (79%) (100). Conversely, other publications suggested that using the G-15 formulation, higher sperm viability could be obtained in thawed buffalo semen (52), G-75 being more effective than G-100 thanks to superior acrosome integrity rate, motility and morphology (101). However, the results of Sephadex filtering may be influenced by the type of buffer that is used, tris citric acid buffers being more appropriate when compared to N-tris-(hydroxymethyl) methyl-2-aminoethanesulfonic acid (TES) or sodium citrate (37).

An additional approach was represented by the use of Sephadex filters enriched with ion-exchangers (76). Comparative use of the conventional G10 filter alone and the up-stated modified technique, at different stages of the freezing cycle, revealed significant differences regarding certain characteristics of the recovered semen such as: mean individual motility, total sperm abnormalities and plasma membrane and acrosome integrity, in favor of the ion-exchanging units (76).

In terms of fertilizing ability, GWF (28.97 ± 4.07 %) and SU (21.33 ± 1.94 %) returned inferior cleavage rates from embryos produced following oocyte insemination with spermatozoa selected after S-G15 filtration (49). Furthermore, when conception rates were analysed, significantly higher results were obtained from buffalo cows inseminated with G-75 filtered semen compared to the use of unfiltered material (83).

Sephadex filtration produced greater total/motile sperm recovery rates compared to swim-up (SU) and better post-filter quality (progressive motility, plasma membrane integrity, viability and cleavage rate) than both swim-up (SU) and glass wool filtration (GWF) in buffalo (49), bovine (99) and boar (72). In terms of fertilization rates (cleavage rate) of *in vitro* matured/fertilized oocytes (73), Sephadex and glass wool filtration (40) yielded better results.

### 2.4 The magnetic-activated cell sorting

The lack of correlation between sperm density and apoptosis in DGC could potentially lead to unsuccessful fertilization. Phosphatidylserine (PS) externalisation to the outer membrane leaflet is the basis for the successful separation of apoptotic and non-apoptotic spermatozoa by MACS employing annexin V-conjugated (a 35-kDa protein) microbeads. To improve the quality and function of sperm, MACS and DGC can be combined as a sperm preparation procedure (102). PS, which is negatively charged and is located on the inner leaflet of the plasma membrane in viable cells, is bound by annexin V. However, early in apoptosis, PS externalises to the outer leaflet of the membrane, a change that is positively correlated with damage to nuclear DNA and has implications for fertilization and pregnancy failure after ART (103).

MACS is performed immediately after DGC in order to evaluate the sperm recovery rate. This rate is calculated by dividing the total motile sperm after MACS by the total motile sperm before MACS (102).

From all the studied combinations of sperm separation techniques within the annexin-negative fraction separated by MACS + DGC the sperm quality is improved and there is very little cell loss (77, 79). This approach had a favorable effect especially on sperm motility and viability, and so, this is why this combination is regarded as a successful sperm preparation method (77, 102). Moreover, this fraction expressed the fewest apoptotic markers (active capase-3, integrity of membrane mitochondrial potential, phospholipid phosphatidylserine) (77). The proportion of sperm DNA fragmentation (9.2 ± 0.7% vs. 12.5 ± 1.0%), mitochondrial membrane potential disruption (18.7 ± 1.9% vs. 27.2 ± 3.0%) and the externalisation of phosphatidylserine (5.9 ± 1.3% vs. 8.2 ± 2.0%) subsequent to MACS was significantly reduced compared to the DGC (78).

The poor rates of fertilization and implantation observed in assisted reproduction may be partially explained by the presence of deregulated apoptosis in spermatozoa (77). Sperm recovery rates in MACS+DGC (73.8 ± 12.1%) was higher than if only DGC (66.7 ± 19.1%) was used (38, 102).

In terms of producing motile, viable, and non-apoptotic spermatozoa, the combination of density gradient centrifugation with annexin-V magnetic cell sorting was superior to all other sperm preparation techniques (101, 102). After MACS + DGC, the rates of sperm recovery were slightly higher (73.83 ± 12.08 vs. 66.67 ± 19.12) than after DGC alone (102). Moreover, using this technique even in teratospermic asthenozoospermic and oligoasthenozoospermic men, the DNA integrity and the functionality will be excellent and it may improve the number of good quality embryos (104, 105).

## 3 Conclusion and prospects

Significant advancements in sperm analysis have been made in the last decade in buffalo reproduction, opening up new paths for subfertility diagnosis and therapy. Traditional sperm sorting methods rely on centrifugation processes, which are known to produce oxidative stress and, as a result, cell damage. Quantitative examination of sperm motility, morphology, and genetics gives useful information for diagnosing male infertility and allowing ideal sperm for ART selection. The various approaches described in this review for buffaloes’ sperm selection have pros and cons, and, as described, several of these methods have yielded contradictory results, and their clinical relevance is therefore still in question. In terms of motility, SU offered a significantly higher MI comparing to DGC, but the recovery rates were generally lower. Additionally, using a MACS and DGC protocol the sperm recovery rates were higher than using only DGC.

The most effective way to increase freezability and cryopreserve low-quality buffalo bull ejaculates is by sperm separation techniques. While in human medicine, these methods are more investigated, in veterinary medicine, there are still some limitations. Even if several studies have been carried out on other species (dog, cattle bull, boar), buffalo things are still not fully elucidated and they deserve to be further researched for a better thoroughness.

Considering these questionable results, we can conclude that semen separation techniques, both in buffaloes and men, are useful tools for reducing fertility problems, but require much more research to enter into common practice.

## Author contributions

CA: Writing – review & editing. FP: Conceptualization, Writing – original draft, Writing – review & editing. NC: Conceptualization, Data curation, Writing – review & editing. IM: Data curation, Supervision, Validation, Writing – review & editing.
